# Takotsubo Cardiomyopathy Secondary to Adrenal Insufficiency: A Case Report and Literature Review

**DOI:** 10.1155/2020/6876951

**Published:** 2020-06-07

**Authors:** Ian Garrahy, Peter Nicholas, Oreoluwa Oladiran, Salik Nazir

**Affiliations:** ^1^Department of Internal Medicine, Reading Hospital and Medical Center, Tower Health, West Reading, PA, USA; ^2^Department of Cardiology, University of Toledo Medical Center, Toledo, Ohio, USA

## Abstract

We report a case of a middle-aged female who presented with altered mental status, hypotension, and hypoglycemia and was diagnosed with secondary adrenal insufficiency. She was also found to have elevated troponin I on initial evaluation with diffuse T wave inversions on electrocardiogram. Transthoracic echocardiogram revealed ejection fraction of 38% with apical akinesia. Subsequent left heart catheterization revealed clean coronary arteries. She was diagnosed with typical Takotsubo cardiomyopathy secondary to adrenal insufficiency. She was managed with IV hydrocortisone with resolution of symptoms. This article adds to the select few cases in the literature of the association of Takotsubo cardiomyopathy resulting from secondary adrenal insufficiency.

## 1. Introduction

Stress-induced cardiomyopathy, also known as Takotsubo cardiomyopathy (TCM), apical ballooning syndrome, and broken heart syndrome, is an increasingly reported form of nonischemic cardiomyopathy characterized by reversible left ventricular dysfunction in the absence of anatomically corresponding angiographically significant coronary artery lesion [1], dyskinesia with hyperkinesia of the midventricular and/or apical segments of the left ventricle (termed “basal ballooning variant” or “reverse TC”), and a focal dyskinesia of any segment of the left ventricle (“focal variant”) [2]. The disorder is a commoner in postmenopausal women and usually precipitated by intense physical and emotional stress [2]. In rare cases, it is reported to be associated with adrenal insufficiency. We aim to report a similar case of TCM associated with adrenal insufficiency and review the existing literature on this association.

## 2. Case Description

A 55-year-old female with a history of eczema on topical steroids was admitted with altered mental status, hypotension, and hypoglycemia. She presented with a blood glucose of 29 mg/dL which was corrected with IV dextrose. She was started on norepinephrine for her hypotension that was unresponsive with crystalloids. On physical exam, blood pressure was 100/67 (78) on norepinephrine and she was lethargic but arousable to sternal rub. The patient reported that she applied desoximetasone 0.25% cream 2 times daily over her whole body for eczema over the past decade.

Labs were remarkable for morning cortisol of 2.8 mcg/dL, ACTH < 5 pg/mL, and sodium 132 mEq/L. The ACTH stimulation test yielded inadequate cortisol responses of 8.1 and 12.0 mcg/dL, before and 90 minutes after the ACTH, respectively. Given these laboratory findings, it was concluded that she had secondary adrenal insufficiency due to her chronic topical steroid use. She was treated with IV hydrocortisone 100 mg every 8 hours which quickly resolved all her electrolyte derangements (including hypoglycemia) and hypotension and improved her encephalopathy.

**Figure 1:**
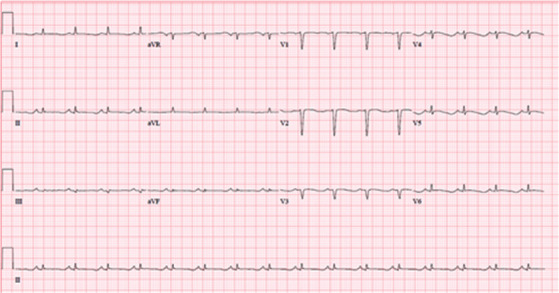
EKG.

**Figure 2:**
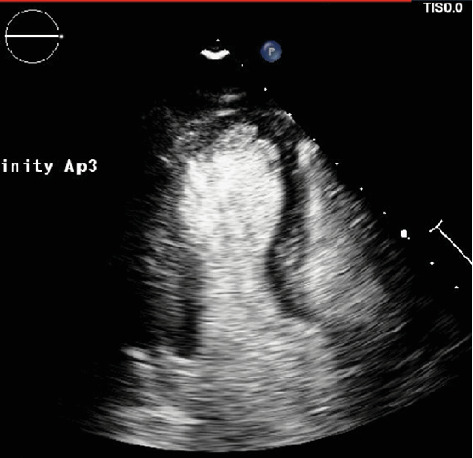
Apical 2 chamber echocardiogram with definity contrast.

**Figure 3:**
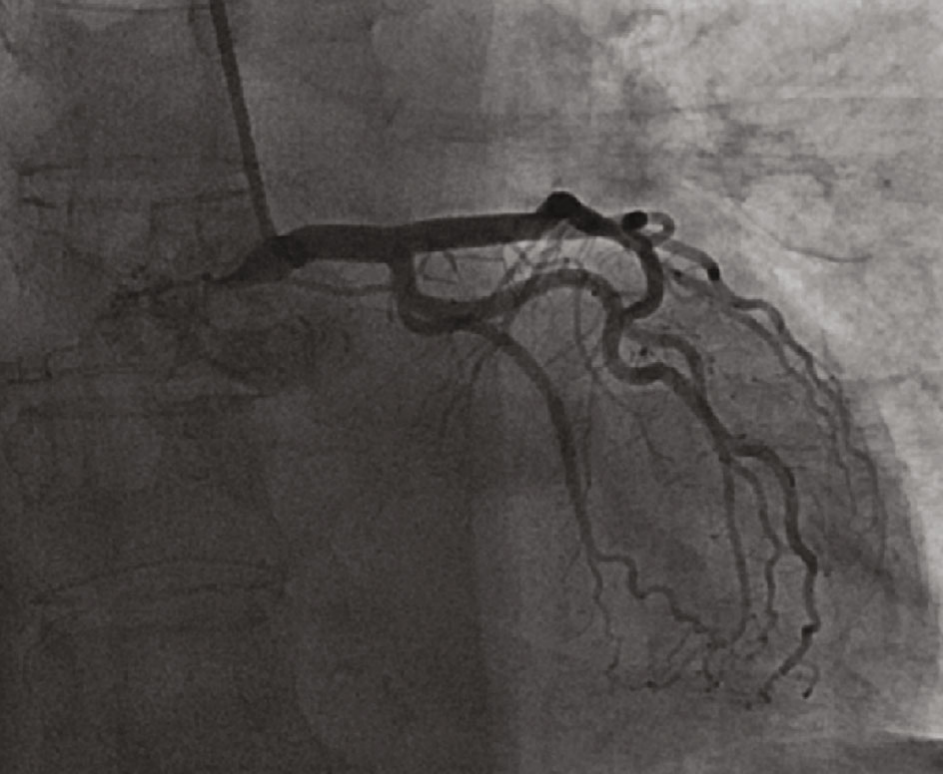
Cardiac catheterization of the left coronary artery.

Concurrently, the patient was noted to have an elevated troponin, which peaked at 0.37 ng/mL. The patient denied chest pain, dyspnea, lightheadedness, or palpitations. Her EKG exhibited diffuse T wave inversions (Figure 1). Transthoracic echocardiogram revealed an ejection fraction of 38% with apical akinesis (Figure 2) concerning for typical TCM. Given that the patient was asymptomatic, we discussed a nuclear stress test versus left heart catheterization with the patient, and it was mutually decided to proceed with a nuclear stress test which demonstrated reversible apical inferior perfusion defect and apical inferior wall motion abnormality. Eventually, the patient underwent a left heart catheterization which showed clean coronaries (Figure 3). Based on these findings, she was diagnosed with typical TCM secondary to adrenal insufficiency due to exogenous steroid use. Her troponin subsequently trended down, her IV hydrocortisone was tapered to oral hydrocortisone, and she was discharged in stable condition with endocrine and cardiology outpatient follow-up.

## 3. Discussion

Patients with Takotsubo cardiomyopathy typically suffer from an acute stressor. In the present case, we attribute the Takotsubo cardiomyopathy to secondary adrenal insufficiency as evidenced by the decreased ACTH, cortisol, and the lack of adequate response of cortisol levels to ACTH administration. Although Takotsubo cardiomyopathy may have been caused by other causes such as the hyponatremia and hypoglycemia, it is more likely that adrenal insufficiency was the cause as it is a unifying diagnosis for hyponatremia, hypotension, and hypoglycemia. We therefore made a diagnosis of Takotsubo cardiomyopathy due to secondary adrenal insufficiency.

Most cases of Takotsubo cardiomyopathy associated with adrenal insufficiency are documented in the pediatric age group [3–5]. To our knowledge, only four cases of Takotsubo cardiomyopathy associated with adrenal insufficiency have been reported in the English literature in the adult patients (Table 1). Three of the four cases were associated with secondary adrenal insufficiency [6–8]. All four cases showed reversible cardiomyopathy and improved myocardial function with steroid therapy for adrenal insufficiency. In the present case, the patient was lost to cardiology follow-up, so there is no evidence for reversibility of our patient's cardiomyopathy. However, the patient's hypoglycemia and hypotension improved quickly with IV hydrocortisone, highlighting the importance of early supplemental hydrocortisone in these patients.

The cause of left ventricular dysfunction in Takotsubo cardiomyopathy is still unknown. It may represent catecholamine-induced myocardial stunning that results from a combination of myocardial ischemia related to diffuse microvascular dysfunction and multivessel epicardial spasm [9]. Although the evidence is strong that catecholamines play a large role in the acute reversible myocardial injury, it is still unclear why the apical segment is more affected than the basal segment [10]. The most plausible explanation is the increased volume of adrenergic receptors in the apex of the heart [11].

While it is known that treatment of the underlying condition/removal of the stressor is the cornerstone for management of TCM which in this case was glucocorticoid therapy, studies have also shown that glucocorticoids are important for the maintenance of membrane calcium transport function in the cardiac sarcoplasmic reticulum and, thus, for myocardial contractility [12]. Glucocorticoids also reduce ischemia-reperfusion-induced myocardial apoptosis [13]. Furthermore, intracellular calcium concentration is affected by glucocorticoids. The authors also showed that microsomal phosphorylase activity in the myocardium was depleted by adrenalectomy [14]. The decreased phosphorylase activity may impair glycogenolysis and the excitation-contraction coupling of the heart [15–17]. Considering all these effects, it can be concluded that glucocorticoids can rescue myocyte apoptosis and improve cardiac function.

This present case reports the rare finding of Takotsubo cardiomyopathy caused by secondary adrenal insufficiency. Treatment of the inciting etiology along with careful monitoring of cardiac function coupled with appropriate guideline-directed treatment of cardiomyopathy can reverse the left ventricular dysfunction.

## Figures and Tables

**Table 1:** Reported cases of adrenal insufficiency-induced Takotsubo cardiomyopathy.

Case	Age/sex	Primary vs. secondary AI	Coronary angiogram	Typical vs. atypical TCM	Author
1	74/F	Secondary	No abnormalities	Typical	Iga et al.
2	64/F	Primary	No abnormalities	Typical	Iga et al.
3	62/F	Secondary	No significant stenosis	Typical	Eto et al.
4	53/F	Secondary	No significant stenosis	Typical	Sakihara et al.
5	55/F	Secondary	No abnormalities	Typical	Garrahy et al.
